# *Notes from the Field:* Multiple Modes of Transmission During a Thanksgiving Day Norovirus Outbreak — Tennessee, 2017

**DOI:** 10.15585/mmwr.mm6746a4

**Published:** 2018-11-23

**Authors:** Julia Brennan, Steffany J. Cavallo, Katie Garman, Kailey Lewis, D.J. Irving, Christina Moore, Linda Thomas, Jeffrey Hill, Raquel Villegas, Joe F. Norman, John R. Dunn, William Schaffner, Timothy F. Jones

**Affiliations:** ^1^Epidemic Intelligence Service, CDC; ^2^Division of Scientific Education and Professional Development, CDC; ^3^Communicable and Environmental Diseases and Emergency Preparedness, Tennessee Department of Health; ^4^Department of Health Policy, Vanderbilt University Medical Center, Nashville, Tennessee.

On November 28, 2017, the manager of restaurant A in Tennessee reported receiving 18 complaints from patrons with gastrointestinal illness who had dined there on Thanksgiving Day, November 23, 2017. Tennessee Department of Health officials conducted an investigation to confirm the outbreak, assess exposures, and recommend measures to prevent continued spread.

On November 23, one patron vomited in a private dining room, and an employee immediately used disinfectant spray labeled as effective against norovirus* to clean the vomitus. After handwashing, the employee served family-style platters of food and cut pecan pie. For the November 23 Thanksgiving Day, restaurant A served 676 patrons a limited menu from 11 a.m. to 8 p.m. The manager provided contact information, seating times, and seating locations for 114 patrons with reservations. All patrons with contact information were telephoned, and a questionnaire was used to assess illness and exposures for anyone living in the household who ate at restaurant A on November 23. Stool specimens were requested from ill patrons. Among the 676 patrons, 137 (20%) were enrolled in a case-control study. 

A probable case was defined as diarrhea (three or more loose stools in 24 hours) or vomiting within 72 hours of eating at restaurant A on November 23; probable cases with norovirus RNA detected in a stool specimen by real-time reverse transcription–polymerase-chain reaction (RT-PCR) were considered confirmed. On November 30, environmental swabs for norovirus testing were collected in the restaurant. Patient and environmental samples were tested by real-time RT-PCR and sequenced at the Tennessee State Public Health Laboratory.

Thirty-six (26%) case-patients (two confirmed and 34 probable) and 101 (74%) controls were enrolled in the case-control study. Illness onsets occurred during November 23–25, with 17 of 35 (49%) cases occurring on November 24 (Figure). The mean incubation period was 31 hours (range = 2.5–54.5 hours), and the mean illness duration was 3 days (range = 0–6 days). Only one case-patient sought medical care. Diarrhea was reported by 33 (94%) case-patients, fatigue by 29 (83%), nausea and abdominal cramps by 28 (80%), vomiting by 24 (69%), and fever by six (17%).

**FIGURE:**
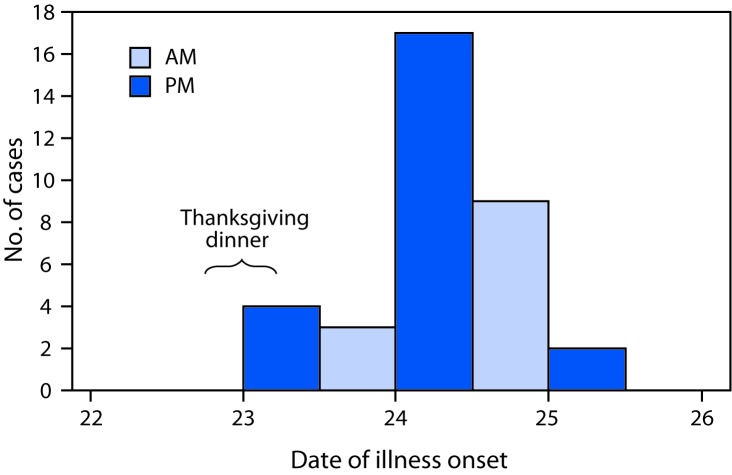
Cases of gastrointestinal illness among patrons of a restaurant — Tennessee, November 2017* * N = 35; onset date was not available for one of the laboratory-confirmed cases in the outbreak.

Among menu items, only pecan pie was significantly associated with illness (odds ratio [OR] = 2.6; 95% confidence interval [CI] = 1.1–5.8); however, it was eaten by only 16 (47%) of 34 case-patients. The vomiting event occurred around noon; patrons seated during 11 a.m.–1 p.m. were significantly more likely to become ill than were patrons seated during other times (OR = 6.0; 95% CI = 2.6–15.3). No significant differences between dining locations (i.e., private dining room versus general seating) were identified (OR = 1.4; 95% CI = 0.4–4.3). Logistic regression was used to evaluate the effects of eating pecan pie, seating time, and seating location; only seating time during 11 a.m.–1 p.m. remained statistically significant (OR = 6.0; 95% CI = 2.2–16.5).

Stool specimens from two case-patients identified Norovirus GII.P16-GII.4 Sydney. Norovirus GII was identified in one environmental swab collected from the underside of a table leg adjacent to the vomitus.

A point-source norovirus outbreak occurred after an infected patron vomited in a restaurant. Transmission near the vomiting event likely occurred by aerosol or fomite. Norovirus spread throughout the restaurant could have occurred by aerosol, person-to-person, fomite, or foodborne routes. Inadequate employee handwashing likely facilitated foodborne transmission through servings of pecan pie.

In hospital settings, CDC and the Tennessee Department of Health recommend contact precautions (e.g., gloves and gowns) when personnel have contact with vomitus (*1*). Similarly, the Food and Drug Administration’s 2017 Food Code recommends restaurants have a written plan detailing when and how employees should use personal protective equipment for cleaning vomitus (*2*). Reinforcing the need for proper handwashing and performing thorough environmental cleaning with appropriate personal protective equipment in food service establishments can prevent or mitigate future outbreaks.

## Footnotes

* Active ingredients = n-Alkyl dimethyl benzyl ammonium chlorides and n-Alkyl dimethyl ethylbenzyl ammonium chlorides.
